# Genome alterations associated with improved transformation efficiency in *Lactobacillus reuteri*

**DOI:** 10.1186/s12934-018-0986-8

**Published:** 2018-09-03

**Authors:** Laura Ortiz-Velez, Javiera Ortiz-Villalobos, Abby Schulman, Jee-Hwan Oh, Jan-Peter van Pijkeren, Robert A. Britton

**Affiliations:** 1 Department of Molecular Virology and Microbiology, Baylor College of Medicine, One Baylor Plaza, Houston, TX USA; 2 Comparative Medicine and Integrative Biology, Michigan State University, East Lansing, MI USA; 3 Department of Cognitive Sciences, Rice University, Houston, TX USA; 4 Department of Food Science, University of Wisconsin-Madison, Madison, WI USA

**Keywords:** *Lactobacillus reuteri* 6475, pSIP411, Transformation efficiency, Genome engineering, Mobile genetic elements, Prophage

## Abstract

**Background:**

Lactic acid bacteria (LAB) are one of the microorganisms of choice for the development of protein delivery systems for therapeutic purposes. Although there are numerous tools to facilitate genome engineering of lactobacilli; transformation efficiency still limits the ability to engineer their genomes. While genetically manipulating *Lactobacillus reuteri* ATCC PTA 6475 (LR 6475), we noticed that after an initial transformation, several LR 6475 strains significantly improved their ability to take up plasmid DNA via electroporation. Our goal was to understand the molecular basis for how these strains acquired the ability to increase transformation efficiency.

**Results:**

Strains generated after transformation of plasmids pJP067 and pJP042 increased their ability to transform plasmid DNA about one million fold for pJP067, 100-fold for pSIP411 and tenfold for pNZ8048. Upon sequencing of the whole genome from these strains, we identified several genomic mutations and rearrangements, with all strains containing mutations in the transformation related gene A (*trgA*). To evaluate the role of *trgA* in transformation of DNA, we generated a *trg*A null that improved the transformation efficiency of LR 6475 to transform pSIP411 and pJP067 by at least 100-fold, demonstrating that *trg*A significantly impairs the ability of LR 6475 to take-up plasmid DNA. We also identified genomic rearrangements located in and around two prophages inserted in the LR 6475 genome that included deletions, insertions and an inversion of 336 Kb. A second group of rearrangements was observed in a Type I restriction modification system, in which the specificity subunits underwent several rearrangements in the target recognition domain. Despite the magnitude of these rearrangements in the prophage genomes and restriction modification systems, none of these genomic changes impacted transformation efficiency to the level induced by *trgA*.

**Conclusions:**

Our findings demonstrate how genetic manipulation of LR 6475 with plasmid DNA leads to genomic changes that improve their ability to transform plasmid DNA; highlighting *trgA* as the primary driver of this phenotype. Additionally, this study also underlines the importance of characterizing genetic changes that take place after genome engineering of strains for therapeutic purposes.

**Electronic supplementary material:**

The online version of this article (10.1186/s12934-018-0986-8) contains supplementary material, which is available to authorized users.

## Background

Advances in the fields of genome engineering and synthetic biology are enabling editing of host resident microbes to understand their effect on health and to explore their therapeutic potential [1, 2]. Lactic acid bacteria (LAB) are a diverse group of microorganisms, including *Lactococcus* sp., and *Lactobacillus* sp., that have been widely used in the fermentation of food and medicine [3–5]. Some LAB are natural inhabitants of the human microbiome and are proposed to provide probiotic benefits to humans, making LAB one of the chassis of choice to build diagnostic tools and therapeutic delivery systems [6–10]. Although there are available genetic tools that allow heterologous gene expression and genome manipulation for a few LAB, such as *Lactococcus lactis*, there is still a need to optimize genome engineering of other biologically relevant LAB [11, 12]. A common factor that impairs genetic manipulation of these bacteria is their limited ability to take up recombinant DNA; therefore, characterizing the mechanisms that impact transformation efficiency of *Lactobacillus* sp. could facilitate the development of approaches that improve transformation efficiency of other lactobacilli [11, 12].

On the other hand, a significant challenge in genetic engineering of non-traditional microorganisms is to limit the number of times these organisms are passaged to reduce mutations that adapt these organisms to the laboratory environment. In model systems such as *Escherichia coli* K-12, long-term passage in the laboratory and selection for qualities that have made this strain genetically tractable has rendered this organism laboratory adapted and unable to compete in the gut environment [13]. Similarly, Renda et al. [14] observed that laboratory manipulation of *Acinetobacter baylyi* ADP1 led to the loss of cell competence to transform DNA due to the re-activation of phage emergence during an experimental evolution study [14, 15].

The pSIP inducible expression system is one of the most widely used vectors to achieve control of gene expression in several *Lactobacillus* spp. in laboratory conditions [16–18]. The pSIP411 expression system contains a broad-host-range, high-copy-number pSH71 replicon with a rolling-circle type replication mechanism, an origin also previously used for the development of the commonly used pNZ vectors [16, 19]. Here we report how genetic manipulation of *Lactobacillus reuteri* 6475 (LR 6475) with plasmids that are widely used in LAB significantly improves transformation efficiency of this strain. To identify the nature of the improvement in the ability to take up plasmid DNA, we performed whole-genome sequencing analysis and a series of experiments that highlighted the gene *trgA* (*t*ransformation *r*elated *g*ene A) as the primary driver of this phenotype. Transformation of LR 6475 with constructs of different efficiencies, (1) improves its ability to take up plasmids, (2) promotes mutations in *trgA*, and (3) generates several chromosomal mutations that are likely to have an impact on bacterial physiology. Overall our findings demonstrate how the transformation of recombinant DNA improves transformation efficiency of LR 6475 and highlights the importance of being vigilant to document genomic and physiological changes as they occur during the engineering of non-traditional microorganisms.

## Results

### Plasmid transformation of LR 6475 generates strains with higher transformation efficiency

**Fig. 1:**
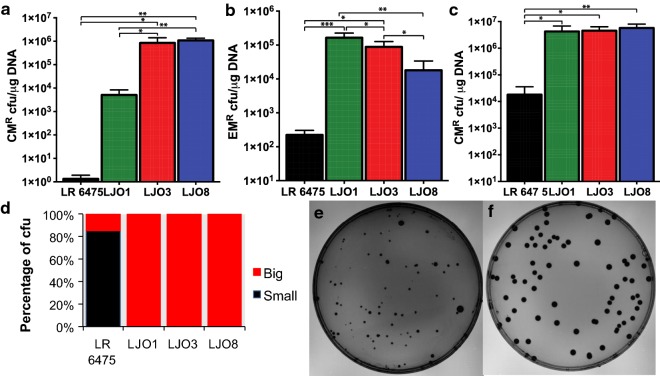
Transformation of pSH71 derivatives increases transformation efficiency of LR 6475. Transformation efficiency of LR 6475 and mutants generated by plasmids transformation. LJO1, LJO3 and LJO8 were cured from their original plasmids and retransformed with pJP067 (**a**), pSIP411 (**b**) and pNZ8048 (**c**). CM^R^: chloramphenicol resistant, EM^R^: erythromycin resistant. Data represents the averages of three independent experiments and error bars represent standard deviation. (*, **, *** indicates the significant difference at p < 0.05 p < 0.01 and p < 0.001, respectively). **d** Measurement of the colony size after transformation of pNZ8048, including LR 6475 (**e**) and LJO3 (**f**). Colony forming Units (CFU) were divided based on their radius as small (0–6 units) and big (7–18 units). Radius was a measure of the pixel size. Data represents the averages of three independent experiments

The high-copy-number lactococcal replicon pSH71 has been widely used to build plasmids for replication in various species of lactobacilli [16, 19]. Different plasmids containing this broad-host-range replicon have dramatically different transformation efficiencies in LR 6475. For example, pJP067 has very low efficiency and pNZ8048 has significantly higher efficiency, despite the fact they contain highly similar replication origins. pSIP411, a commonly used vector for inducible gene expression in *Lactobacillus*, and the pSIP411 derivative pJP042 have an intermediate level of transformation efficiency [16, 18]. While generating additional tools for genetic engineering of LR 6475, we noticed that strains previously transformed with pJP042 and pJP067 had improved the ability to take up plasmids that were typically poorly transformed into LR 6475 (Fig. 1). These strains, named LJO1, LJO3 and LJO8, were generated by the transformation of pJP042 (LJO1) or pJP067 (LJO3 and LJO8) into LR 6475.

To confirm that LJO1, LJO3, and LJO8 had increased transformation efficiency, we cured them of the plasmid and re-transformed these strains with plasmids pNZ8048, pSIP411 and pJP067 (Fig. 1). Each mutant strain displayed improved ability to take up DNA, ranging from one to six orders of magnitude when compared to LR 6475. The change in transformation efficiency was most dramatic when strains were transformed with pJP067, representing a 1000-fold increase in LJO1 and a 100,000-fold increase in LJO3 and LJO8 compared to LR 6475 (Fig. 1a). Since pJP042 encodes the RecT recombinase, which we did not want to be a confounding factor in the study, we used the parental pSIP411 vector. When this vector was transformed into the mutant strains we found a 50- to 100-fold increase in transformation efficiency compared to wild-type LR 6475 (Fig. 1b). A similar result was observed for pNZ8048, in which all mutants had a transformation efficiency of two orders of magnitude higher than LR 6475 (Fig. 1c). Interestingly, LJO3 and LJO8 are 100 times more efficient at transforming pJP067 than LJO1; whereas pSIP411 achieves similar efficiencies among all strains. This finding suggests that the factors that restrict pSIP411 are equally inactivated in the LJO strains, whereas pJP067 is possibly restricted by different factors and those factors are inactive in LJO3 and LJO8, but are still partially active in LJO1.

We also noticed that the LJO strains displayed larger colony sizes compared to the wild-type strain LR 6475 (Fig. 1d–f) after transformation, supporting the hypothesis that transformation of plasmids in LR 6475 lead to bacterial adaptations that improve its ability to harbor recombinant DNA.

### Genomic changes induced by plasmid transformation

To identify the mechanisms responsible for the increase in transformation efficiency, we analyzed the genomes of mutant strains using Illumina sequencing technology. We initially identified a group of mutations located in the gene LAR_0821 (hereafter called *trg*A for *t*ransformation *r*elated *g*ene A). This gene has been annotated as a hypothetical protein (IMG database, Integrated Microbial Genomes & Microbiomes database) and is located directly downstream of a Type I Restriction-Modification (RM) system (denoted here LreRMI). TrgA is found in several *Lactobacillus* species in addition to *L. reuteri* (including *L. crispatus*, *L. salivarius*, *L. kefiranofaciens* and *L. timonensis*) although it is not universally conserved among all lactobacillus. Outside the *Lactobacillus* genus, only the bacterium *Chlamydia trachomatis* has a gene with clear homology to *trgA*. From the genome analysis of the LJOs stains, we identified a missense mutation in *trg*A (P584T) in strain LJO1 and two consecutive missense mutations in *trg*A in strain LJO8 (G411R, K412R); whereas LJO3 deleted the entire *trg*A sequence along with the LreRMI locus.

To completely close the genomes and characterize any other rearrangements that may have occurred, we utilized Pacbio Smart sequencing technology, which identified two additional genomic changes within the strains displaying the higher transformation efficiency phenotype. One of these changes included a series of genome rearrangements that occurred around a region flanked by two active prophages present in the LR 6475 genome. The second group of genome alterations happened in a second Type I RM system locus (named LreRMII) in which the sequence of the specificity subunit genes was rearranged compared to LR 6475. Since there were three groups of mutations identified by whole-genome sequencing that were present in the mutant strains, we individually investigated the association between these mutations and improvement in transformation efficiency.

### Plasmid transformation induces mutations in *trgA*

Since all LJO strains acquired mutations in the *trgA* gene, we hypothesized this gene was a key factor limiting transformation efficiency in LR 6475. We initially sought to confirm the association between the transformation of plasmid DNA and the occurrence of mutations in *trgA*. For this purpose, we transformed pSIP411, pJP042 and pNZ8040 into LR 6475 and evaluated the frequency of mutations induced in *trgA*. We screened for *trgA* mutations present in a total of 23 transformants per plasmid, identifying eight mutations for pSIP411, five mutations for pJP042, and only one mutation for pNZ8048 (Table 1). All mutations were different from the missense mutations initially observed for LJO1 and LJO8, and were mostly located towards the 3′ end of the gene (Table 2). Interestingly, 62.5% of the mutations generated by the transformation of pSIP411 were stop codons (5/8), whereas all mutations obtained with the transformation of pJP042 were missense mutations; suggesting that pSIP411 induces more pressure on the cell to abolish the activity of *trgA*. This finding is congruent with the mutation frequency and the types of mutations observed for pJP067, a plasmid with very low transformation efficiency for which all transformants have evidenced mutations in *trgA*. No mutations were found in the strains transformed with water or single-stranded DNA. The generation of mutations in *trg*A suggests that transformation of plasmids into LR 6475 frequently drives the modification of *trg*A activity to facilitate uptake of these plasmids, and thus likely plays a role in transformation efficiency.

**Table 1:** Frequency of mutants generated by transformation of pSH71 derivative plasmids

Plasmid	Number of colonies with mutations in *trg*A	Number of colonies screened	Proportion of mutants
pSIP411	8	23	0.34
pJP042	5	23	0.22
pNZ8048	1	23	0.04
pJP067	2	2	1

**Table 2:** Description of *trg*A mutations generated in LR 6475 when transformed with pSH71 derivatives

Plasmid	DNA mutation position	Base reference	SNP	Amino acid ref *trg*A	Amino acid mutation
pJP042	490	C	A	Glutamine	Lysine
	1283	G	A	Glycine	Aspartic acid
	1506	G	C	Methionine	Isoleucine
	1750	C	A	Proline	Threonine^a^
	1991	G	A	Glycine	Glutamic acid
	2004	G	C	Methionine	Isoleucine
pSIP411	598	Deletion of 202 bp			
	928	C	T	Glutamine	Stop codon
	1223	C	T	Serine	Leucine
	1534	C	A	Glutamine	Lysine
	1564	C	T	Arginine	Cysteine
	1838	C	A	Serine	Stop codon
	1915	C	T	Glutamine	Sop codon
	2099	G	A	Tryptophan	Stop codon
pJP067	1231, 1235	G, A	A, G	Glycine, lysine	Arginine, arginine^b^
	Completion deletion of the gene^c^				
pNZ8048	80	A	G	Glutamic acid	Glycine

^a^LJO1 mutation

^b^LJO8 mutation

^c^LJO3 mutation

### Mutations in *trgA*, but not in LreRMI, are sufficient to improve transformation efficiency of LR 6475

**Fig. 2:**
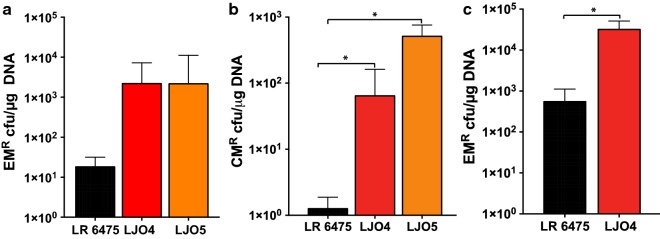
Role of *trg*A and LreRMI in the higher transformability phenotype observed in *LR* 6475. Transformation efficiency of *LR* 6475, LJO4 (*trg*A null mutant) and LJO5 (the double null mutant for *trg*A and the predicted restriction unit of LreRMI, [LAR_0819]) for **a** pSIP411 and **b** pJP067. **c** Transformation efficiency of LR 6475 and the *trg*A null mutant for pSIP411 isolated from *LR* 6475. CM^R^: chloramphenicol resistant, EM^R^: erythromycin. Data represents the averages of three independent experiments and error bars represent standard deviation. (*indicates the significant difference at p < 0.05)

Based on Basic Local Alignment Search Tool (BLAST) analysis, *trgA* encodes a hypothetical protein of 755 amino acids with distant homology to the N-terminus of the catalytic domain of BfiI and NgoFVII, which are type II restriction endonucleases (Additional file 1: Figure S1a). Directly upstream of the *trgA*, there is a cluster of genes annotated as part of a type I RM system (LreRMI) (Additional file 1: Figure S1b). This finding, coupled with the dependency on plasmid transformation to induce mutations in *trgA*, led us to hypothesize that mutations in *trgA* improve the ability of LR 6475 to take up plasmid DNA by inactivating the activity of the LreRMI. To test this hypothesis, we generated a single null mutant for *trg*A (LJO4) and a double mutant containing null mutations in *trg*A and the predicted restriction endonuclease present in LreRMI (LAR_0819); this strain is named LJO5. We then compared their transformation efficiencies for pSIP411 and pJP067 into LJO5 with the efficiency of LR 6475 (Fig. 2a, b). Both strains had similar transformation efficiencies for pSIP411, approximately 100-fold higher than LR 6475 (Fig. 2a). pJP067 showed a similar increase in transformation in both strains compared to LR6475, with LJO5 trending to have a higher transformation efficiency than LJ04. However, this increase was not statistically significant. These data demonstrate that a null mutation in *trg*A is sufficient to improve the ability of LR 6475 to transform pSIP411 and pJP067. These results also indicated that abolishment of the restriction activity of LreRMI in the *trg*A mutant does not have a significant impact on transformation efficiency for either plasmid.

Since we could not purify TrgA to evaluate its role in DNA restriction, we attempted to determine if *trgA* is involved in DNA restriction-modification by transforming host-methylated DNA into LR 6475. We isolated pSIP411 from LR 6475 and then transformed it into LR 6475 and the *trgA* null mutant (LJO4) (Fig. 2c). Transformation of DNA purified from LR 6475 does not significantly improve transformation efficiency of pSIP411 in LR 6475 to the levels achieved by LJO4, suggesting either *trg*A does not directly impact restriction-modification of DNA, or that modification of plasmid by the host does not play a role in transformation efficiency in LR 6475.

### Mutations in *trg*A impact colony size of transformed strains

Based on the observation that wild-type colonies were quite uneven in size after transformation of plasmid DNA whereas the LJO strains were large and uniform (Fig. 1d–f); we hypothesized that colonies with a larger size after transformation would have improved ability to take up plasmid DNA due to mutations in *trgA*. To test this assumption, we transformed plasmids with high (pNZ8048) and medium (pJP042) transformation efficiencies into LR 6475 and selected big and small colonies after transformation. We cured them of the plasmid and re-transformed them with either pJP067 and pNZ8048 to evaluate changes in transformation efficiency. Among the colonies previously transformed with pNZ8048, only one big isolate (12.5%, B3) acquired the phenotype of being able to transform pJP067 (Additional file 2: Figure S2a) as well as yield uniform, large colonies when transformed with pNZ8048. Sequencing of *trg*A in these 8 strains showed that only this single colony acquired a mutation in *trgA*, linking *trgA* function to the acquired phenotypes. For the clones isolated after transformation of pJP042, 7 out of 10 isolates (70%) gained the ability to host plasmid pJP067, with varying degree of efficiencies ranging from 10^2^ to 10^5^ cfu/µg DNA (Additional file 2: Figure S2b). From this group, only two clones, one big (B2) and one small (S2), harbored mutations in *trg*A. The fact that not all strains with high transformation efficiency for pJP067 acquired mutations in *trgA*, suggests that mutations in *trg*A are sufficient, but are not the sole factor that has a role in the improvement in transformation efficiency observed after transformation of plasmid DNA. Taken together, these data further support that mutations in *trgA* improve the transformation efficiency of LR 6475. Additionally, it indicates that other changes, different form *trg*A mutations, are taking place into these strains that allow them to improve transformation of DNA with varying levels of efficiency.

### Transformation of plasmids in LR 6475 promotes prophage-associated genomic rearrangements

**Fig. 3:**
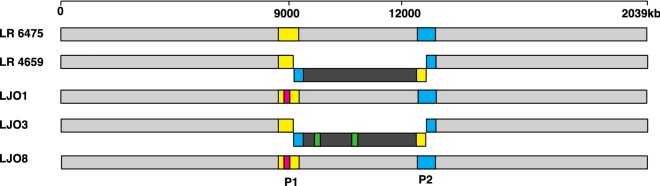
Pairwise genome alignments comparing the genomes of LR 6475 and strains with higher transformation efficiency. Whole genome comparison showing areas that are conserved across genomes in light gray color. The alignment evidences genomic modification sequences located inside or around two LR 6475 prophages (P1, phage 1, yellow; P2, phage 2, cyan). Green areas represent deleted regions in LJO3 that took place in two different locations inside the inverted region and are 34 kb and 48 kb in size. Pink areas represent an insertion (17 kb) in the prophage one present in LJO1 and LJO8, which contains a phage integrase protein among other genes. The dark gray area flanked by P1 and P2, represents the genomic inversion observed in LR 4659 and LJO3 that also flipped part of the prophage genomes. The alignment was done using the Mauve method from the MegAlign Pro application (DNASTAR, Madison, WI)

**Fig. 4:**
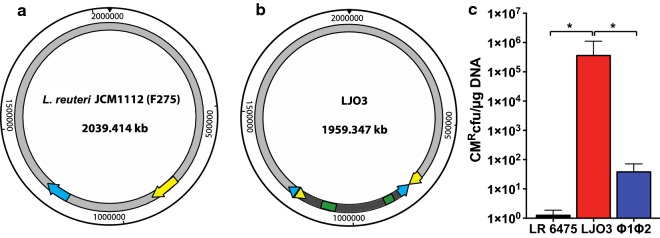
Transformation of pSH71 derived plasmids induces significant genome remodeling in LR 6475 prophages. Chromosome representation of **a** *L. reuteri* JCM112 (F275) and **b** LJO3, representing identical genomic sequences (light gray), inverted (dark gray) and deleted (green) regions. The prophages present in LR 6475 genome are represent in yellow (phage 1) and cyan (phage 2). Arrows indicate the sense of the strand. **c** Transformation efficiency of LR 6475, LJO3 and LR 6475 double phage mutant (∆Φ1∆Φ2) for pJP067. Data represents the averages of three independent experiments and error bars represent standard deviation, (*p < 0.05)

In addition to the mutations present in *trgA*, we also observed additional mutations in or around genomic regions where two active prophages are located. The most striking changes occurred in the LJO3 strain, where one large inversion and two large deletions took place. The inversion arose in a chromosomal area of 336 kb, flanked by two repetitive sequences of 5.3 Kb (Figs. 3, 4a, b) present in the genome of two different LR 6475 prophages. Interestingly, this genomic inversion was also present in LR 4659; another non-lab adapted *L. reuteri* strain that is closely related and has similar transformation efficiencies to LR 6475 (Fig. 3). Because LR 6475 and LR 4659 have comparable transformation efficiencies, we suspected the genome inversion itself does not contribute to the improvement in transformation efficiency.

Activation of phage replication has been shown to impair competence of bacteria to take up DNA [14, 15]. To determine if the rearrangements of the prophages modified the activity of phages and subsequently altered transformation efficiency of the LJO strains, we evaluated the activation of phages after induction with mitomycin C in wild-type and several LJO mutants. We did not find any significant differences among the LJO mutants that could explain the changes observed in transformation efficiency when compared to LR 6475 (Additional file 3: Figure S3). To completely rule out the link between phage activity and the ability to take up plasmid DNA, we evaluated the transformation efficiency of an LR 6475 strain where both prophages were deleted (Fig. 4c). While deletion of LR 6475 prophages slightly improved transformation efficiency of LR 6475, it did not improve to the levels observed for the LJO mutant strains.

### Genomic rearrangements of the LreRMII locus do not directly contribute to improvements in transformation efficiency

**Fig. 5:**
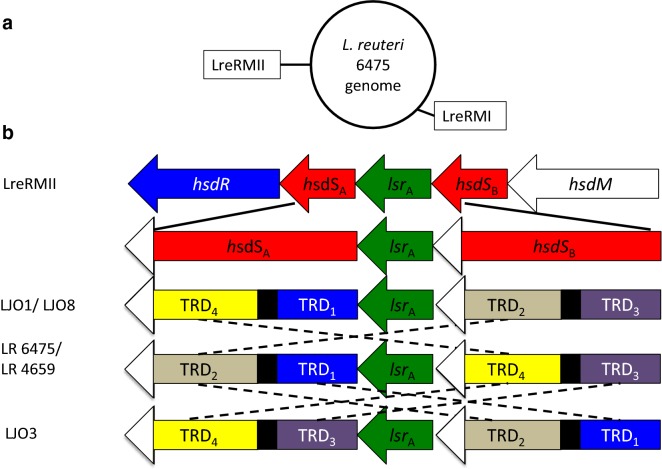
Genomic rearrangements of the specificity unit (HsdS) in the LreRMII locus. **a** Genomic location of the two *L. reuteri* Type I RM systems described in this study. **b** Structure and comparison of the organization of the hsdS units and their predicted target recognition domains (TRDs) in LR 6475, LR 4659 and the LJO strains. Black areas represent repetitive region (CR1); whereas white arrow heads represent conserved sequence among all strains (CR2)

The last group of chromosomal changes found in the LJO strains that may impact transformation efficiency of LR 6475 was a series of rearrangements observed in a second Type I RM system (LreRMII) (Fig. 5). The LreRMII locus consists of five genes, four of which include the common genes for a Type I RM system. The first and the last gene encode for the restriction unit (*hsd*R, LAR_1343) and the methyltransferase unit (*hsd*M, LAR_1347), respectively (Fig. 5b). Two genes encode for the specificity subunit of the system (*hsd*S_A_, LAR_1344; *hsd*S_B_, LAR_1346), which are separated by a gene predicted to be an integrase/recombinase (LAR_1345). We named this gene (*lsrA*) lactobacillus site-specific recombinase A, due to its homology to site-specific recombinases.

We noticed that each of the two *hsdS* genes contained two conserved regions (CR1 and CR2) and two large variable regions (about 500bps) that were arranged differently among the LJO strains (Fig. 5b). Protein function prediction of the specificity unit by I-TASSER (Iterative Threading ASSEmbly Refinement) revealed that the sequences subjected to rearrangements are predicted to be the target recognition domains (TRD1 and TRD2) of the subunits. TRDs are important domains for the recognition of the DNA target sequences and are required for both methylation and restriction of the RM system; therefore, even subtle mutations can lead to significant variations in the DNA target sequence [20]. To confirm that TRDs in LreRMII were rearranged after transformation of plasmid DNA, we sequenced *hsd*S_A_ and *hsd*S_B_ subunits of the ten strains that were previously generated by transformation of pJ042 and improved their ability to transform pJP067 (Additional file 2: Figure S2 and Table 3). Five clones did not modify the structure of the specificity unit after transformation with pJP042, and neither did the transformation negative control (data not shown). The rest of the isolates had different rearrangements of the HsdS subunit, indicating that TRDs were altered after transformation with pJP042. However, we did not find any *hsdS* organization that was associated with a higher transformation efficiency phenotype (Table 3). These data indicate that rearrangements in the specificity units of LreRMII are not directly linked to the changes in transformation efficiency observed after transformation of plasmids in LR 6475.

**Table 3:** Transformation efficiency for pJP067 and organization of the specificity unit *hsds*_*A*_ ans *hsds*_*B*_ in mutants generated after transformation of pJP042

Strain	HsdSA		HsdSB		Transformation efficiency (TE) for pJP067	SD TE of pJP067
	TRD at 5′	TRD at 3′	TRD at 5′	TRD at 3′		
LR 6475	TRD1	TRD2	TRD3	TRD4	1.33E+00	0.58
LR 4659	TRD1	TRD2	TRD3	TRD4	1.00E+00	1
LJO1	TRD1	TRD4	TRD3	TRD2	5.10E+03	3.27E+03
LJO8	TRD1	TRD4	TRD3	TRD2	8.47E+05	2.61E+05
LJO3	TRD3	TRD4	TRD1	TRD2	1.09E+06	5.62E+05
Clone B1	TRD3	TRD2	TRD1	TRD4	0.00E+00	0
Clone B2	TRD1	TRD2	TRD3	TRD4	1.50E+03	8.89E+02
Clone B3	TRD1	TRD4	TRD3	TRD2	2.23E+04	1.41E+04
Clone B4	TRD1	TRD2	TRD3	TRD4	8.33E+03	3.66E+03
Clone B5	TRD1	TRD2	TRD3	TRD4	0.00E+00	0
Clone S1	TRD1	TRD4	TRD3	TRD2	0.00E+00	0
Clone S2	TRD3	TRD2	TRD1	TRD4	5.00E+01	26.46
Clone S3	TRD1	TRD2	TRD3	TRD4	7.27E+02	5.36E+02
Clone S4	TRD3	TRD4	TRD1	TRD2	1.35E+05	8.32E+04
Clone S5	TRD1	TRD2	TRD3	TRD4	7.31E+02	4.19E+02

### Genetic variation of the specificity subunit of LreRMII does not abolish the ability of the system to restrict DNA

**Fig. 6:**
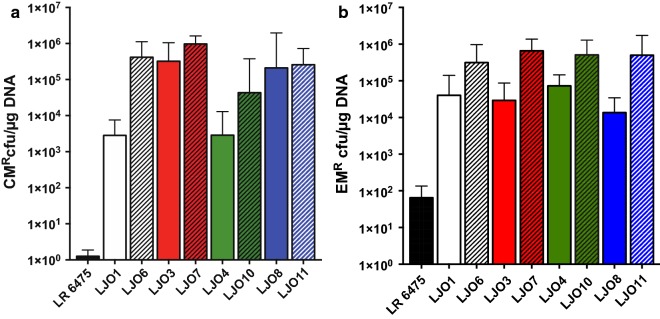
Genomic rearrangements of the LreRMII locus are not directly associated to improvements in TE. Transformation efficiency (TE) of LR 6475 and LJO strains and mutants of the HsdR unit of LreRMII (LAR_1343) for pJP067 (**a**) and pSIP411 (**b**), CM^R^: chloramphenicol resistant, EM^R^: erythromycin resistant. Data represents the averages of three independent experiments and error bars represent standard deviation

Although the organization of the specificity unit in LreRMII was not directly associated with higher transformation efficiency, it was still possible that these changes altered the activity of the RM system. Therefore, we evaluated if rearrangements in the specificity unit abolished the activity of LreRMII in the LJO strains. For this purpose, we generated null mutants for the restriction unit of this RM system (HsdR, LAR_1343) in the LJO mutant backgrounds. We evaluated the capacity of these mutants to restrict foreign DNA by measuring the transformation efficiency for pJP067 (Fig. 6a) and pSIP411 (Fig. 6b). In the case of LJO3 and LJO8, strains that efficiently transform pJP067, inactivation of *hsdR* did not improve their ability to transform this plasmid as shown for LJO7 (LJO3::LAR1343) and LJO11 (LJO8::1343). LJO6 (LJO1::LAR_1343) and LJO10 (LJO4::LAR_1343) displayed a significant increase in transformation efficiency of ~ 100-fold (Fig. 6a). This differential activity was not observed when pSIP411 was transformed. For this plasmid, transformation efficiency was similar across all strains and the efficiency was improved tenfold when the *hsdR* unit was inactivated. Overall, these data evidence that LreRMII is still active in the LJO strains and modification of the *hsd*S unit does not entirely abolish the ability of the RM system to restrict DNA.

## Discussion

Genetic manipulation of human-associated, non-model microorganisms enables the understanding of their impact on host health and identify novel applications for the prevention and treatment of human disease. A critical factor that limits the genetic manipulation of such microorganisms is their ability to take up DNA. Here we describe how genetic manipulation of LR 6475 with constructs commonly used to genetically manipulate LAB induces several genomic alterations that result in improved transformation efficiency. Although we were unable to determine the precise molecular mechanisms that lead to increased transformation, we identified a gene (*trgA*) as one of the primary drivers of improved transformation. Since *trgA* is a highly conserved gene in several lactobacilli, understanding how *trgA* restricts DNA would allow the establishment of strategies to increase transformation efficiency of other *Lactobacillus* species.

One of the main limitations we had in attempting to understand how *trg*A impacts transformation is the fact that the methodologies we use to generate LR 6475 mutants rely on the transformation of low efficiency plasmids (pJP042), and plasmid transformation itself modifies the ability of LR 6475 to transform DNA (Additional file 2: Figure S2). The fact that the mutants generated by recombineering always had mutations in *trg*A, suggests that these strains are also more competent to take up single-stranded DNA; however, we did not find a link between *trg*A and recombineering. Although not all strains that acquired a higher transformation efficiency phenotype had mutations in *trgA*, all *trgA* mutants displayed higher transformation efficiency, demonstrating the association between this gene and the ability to uptake plasmid DNA.

The occurrence of mutations in *trgA* during transformation seems to be inversely related to the transformation efficiency of the plasmid, with a higher proportion of mutants for plasmids that are poorly transformed by LR 6475. These results, combined with the similarity of TrgA to proteins that are involved with restriction modification, suggest that this protein participates in restricting incoming plasmid DNA. We attempted to purify TrgA to test this hypothesis, but unfortunately, the protein was insoluble and could not be purified in an active form. The fact that plasmids isolated from LR 6475 do not improve transformation efficiency in LR 6475 compared to the *trg*A null mutant (LJO4) suggests that modification of DNA by a host mechanism does not play a role in TrgA restriction. However, we cannot rule out that TrgA both restricts and modifies plasmid DNA. We could not establish if *trgA* limits replication of plasmids autonomously or in coordination with LreRMI, since we were not able to generate single null mutants in other genes without acquiring mutations in *trgA*. However, the fact that LJO5 showed a tenfold higher efficiency to transform pJP067 compared to LJO4, suggests that LreRMI is active in the null *trg*A mutant and plays a role in the restriction of pJP067.

We also describe large genetic rearrangements that took place after transformation of plasmid DNA. These mutations were only detected by deep sequencing and by the generation of closed genomes; highlighting the importance of this type of characterization when engineering non-lab adapted strains. It is not clear how these chromosomal mutations and arrangements, such as the ones observed in the genome of LJO3, took place in this strain. However, it is known that transformation of foreign DNA can activate prophages and mobile genetic elements that may have mediated recombination between the two large inverted repeats present in *L. reuteri* prophages, to generate a large genomic inversion. These rearrangements are an example of how genetic manipulation of non-traditional microorganisms may lead to profound genomic arrangements that can impact the physiology and function of a particular bacterium. The type of genomic changes will be determined by the genomic stability, the activity of mobile elements, and the presence of phage or antigenic variation of the strain being manipulated [21–23]. Nonetheless, we did not find a link between the activity of prophages and the improvement in transformation efficiency in LR 6475.

Finally, we also identified a Type I RM system (LreRMII) that shows DNA rearrangements and generates allelic variants for the specificity subunits. These rearrangements are probably mediated by the recombinase *lsrA* and two inverted repeats present in both specificity genes of LreRMII (*hsdS*). We did not find any evidence of a direct association between these rearrangements and the improvement in transformation efficiency or any other physiological change in LR 6475. However, allelic variation of the specificity unit of Type I RM systems have been associated with variations in DNA sequence specificities, DNA methylation or even phase variation and pathogenesis; these changes can significantly impact the interaction between bacteria and their hosts [20, 24–26]. It is not clear why LreRMII appears to be already inactivated in LJO3 and LJO8 for restriction of pJP067, but It is possible that transformation of pJP067 is also limited by factors different from the restriction modification activity of LreRMII, and that these factors are already inactivated in LJO3 and LJO8. Taken together these data suggest that the rearrangements observed in the specificity unit do not abolish the activity of the LreRMII, and it is not likely to be directly associated to the higher transformation efficiency phenotype observed in the LJO strains.

## Conclusions

We demonstrated how genetic manipulation of LR 6475 with plasmid DNA leads to the generation of strains with improved transformation efficiency by inducing several genomic changes, highlighting the gene *trgA* as the primary drivers of this phenotype. Investigating how *trg*A restricts transformation of plasmid DNA might yield relevant knowledge to improve our ability to genetically modify *Lactobacillus* spp. Our findings also emphasize the significance of doing deep genome sequencing to generate de novo, closed genomes for strains that are being engineered with therapeutic purposes. Characterization of this genetically manipulated strains, in both laboratory and native conditions, will be relevant to understand not only the impact of genome engineering on chromosomal modifications but also in cell physiology, its interaction with the host, its ability to colonize a niche or even its pathogenic potential.

## Methods

### Bacterial strains and media

Strains used in this study are listed in Additional file 4: Table S1. *L. reuteri* strains were cultured anaerobically at 37 °C in de Man Rogosa Sharpe (MRS) broth (Difco, BD BioSciences) or on MRS agar plates (1.5% Difco agar). *Lactococcus* *lactis* was grown statically at 30 °C in M17-broth (Difco, BD BioSciences), and supplemented with glucose to a final concentration of 0.5% (w/v). Antibiotics were added to the media, when required, at a concentration of 5 µg/ml of erythromycin and 5 µg/ml of chloramphenicol for lactobacilli and *L. lactis*.

### Reagents and enzymes

All restrictions enzymes were purchased from New England Biolabs (NEB, USA), whereas the lysozyme from chicken egg white and mutanolysin from *Streptomyces globisporus* were purchased from Sigma-Aldrich (Sigma-Aldrich, USA). Phusion polymerase (NEB, USA) was used to generate PCR amplicons for Sanger sequencing, whereas *Taq* DNA polymerase (Denville Scientific, USA) was used for screening purposes. The oligonucleotides used in this study are listed on Additional file 5: Table S2 (Integrated DNA Technology-IDT, USA).

### Plasmid DNA

*Lactococcus lactis* MG1363 was used as the cloning host and source of plasmids used in this study; described in Additional file 4: Table S1 and Additional file 5: Table S2. These plasmids are derived from the pSH71 vector; having all similar origins of replications (ORI). Sequencing and comparison of the origins of these plasmids did not reveal significant differences, other than 3 SNPs and a 62 base pair region, which is not present at the 3′ end of pNZ8048 ORI. pSIP411, pJP042 and pJP067 plasmids have the same ORI sequence. LR 6475 was also used as host source of plasmid when indicated in “Results” section.

### Plasmid isolation from *L. lactis* MG1363 and LR 6475

To isolate plasmids from *L. reuteri*, cultures of 100 ml were grown to an Optical Density (OD) of 1, harvested by centrifugation, washed twice with 0.1 volumes of SET buffer (O.1M NaCl, 10 mM Tris HCl, 1 mM EDTA) and re-suspended in 0.05 volumes of lysis buffer [6.7% sucrose, 50 mM Tris/HCl, 1 mM EDTA)]. Lysozyme and mutanolysin were added to the cells at a concentration of 1 mg/ml and 10 U/ml, respectively. The mixture was incubated at 37 °C for an hour with intermittent shaking. Cells were pelleted by centrifugation and re-suspended in re-suspension solution from the Wizard^®^ Plus SV Miniprep DNA Purification System (Promega, USA); followed by purification of plasmid DNA according to manufacturer’s instructions. Similarly, to isolate plasmids from *L. lactis*, cultures of 10 mls (inoculated with the strain harboring the desired plasmid) were grown for 16 h and washed with 0.1 volumes of TSH buffer (0.7 M sucrose, 30 mM Tris HCl, 3 mM MgCl_2_). The suspensions were incubated with lysozyme (1 mg/ml) for 60 min, followed by plasmid isolation with the Wizard^®^ Plus SV Miniprep DNA Purification System as described for LR 6475.

### Transformation of *L. reuteri*

*Lactobacillus reuteri* and the mutant strains were transformed by electroporation as previously described [27, 28]. Briefly, bacteria are grown until cultures reach an OD_600_ between 0.7 and 0.9, and transformed with 1 μg of plasmid DNA. Cells are recovered for 3 h in one ml of MRS (at 37 °C without shaking) and then plated on media with the appropriate antibiotic selection. To plasmid cure strains, bacteria are grown on broth media without antibiotic for two generations, followed by replica plating on non-selective and selective MRS agar plates to identify colonies that lost the plasmid. Plasmid transformation efficiency was expressed as colony-forming units (cfu) per µg of DNA.

### Analysis of the cfu size

To classify colonies according to their size, we used the Open-source software OpenCFU to determine the radius of the colony [29]. The radius was calculated by counting the number of pixels per colony in images taking from plates containing the *L. reuteri* colonies. *L. reuteri* cells were plated after plasmid transformation on MRS plates, containing the appropriate antibiotic, and were incubated for 48 h at 37 °C. The images were taken with the Alphaimager imaging system (ProteinSimple; California, USA), from plates with a cell density of approximately 7*10^2^ to 1*10^3^ cfu/ml. Colonies were classified as small when cfu radius was between 0 and 6 or as big when the radius was between 7 and 18.

### Genome isolation, sequencing and assembly

Lactobacilli genomes were isolated as previously described based on phenol: chloroform DNA extraction [30]. Purified genomic DNA for each strain was submitted to DNA Link (San Diego, USA) to be sequenced with Illumina or PacBio single molecule, real-time (SMRT) sequencing technology. For PacBio, the SMRTbell library was constructed with SMRTbell™ Template Prep Kit 1.0 (PN 100-259-100) following manufacturer’s instructions (Pacific Biosciences). Smaller fragments, lower than 20 kilobases of SMRTbell template, were removed using Blue Pippin Size selection system for the large-insert library. The library was sequenced using 1 SMRT cells (Pacific Biosciences) using C4 chemistry (DNA sequencing Reagent 4.0) and 240-min movies were captured for each SMRT cell using the PacBio RS II (Pacific Biosciences) sequencing platform [31]. Genome sequencing was performed using 1 SMRT cell with MagBead OneCellPerWell v1 Protocol. De novo assembly was conducted using the hierarchical genome assembly process (HGAP, Version 2.3) workflow [32], including consensus polishing with Quiver. To generate a circular genome, the structure of each contig was verified using MUMmer 3.5 [33] and used to trim one of the self-similar ends for manual genome closure.

Genome analysis was performed using Seqman Pro, MegAlign and Seqbuilder modules of the Lasergene software (DNASTAR, Madison, WI, USA) using the *L. reuteri* JCM1112 genome (F275) as a reference. The sequences of the prophages present in the genome of this strain were determined based on genome analysis of the attachment sites for these prophages (unpublished observations, Jan-Peter van Pijkeren).

### Analysis of *trg*A and restriction enzyme homologs

PSI-BLAST was used to identify possible homologs of *trg*A (Genebank accession number WP_003667956) with a standard p-value cut-off of 0.005. The Integrated Microbial Genomes and Microbiome samples (IMG/M) (https://img.jgi.doe.gov) database was used for analysis of gene neighborhood regions and sequence analysis in *L. reuteri;* using *L. reuteri* F275 or *L. reuteri* JC1112 as reference. The I-TASSER server was used to analyze and predict the structure of *trg*A, as well as the specificity units (*hsd*S) of LreRMII (https://zhanglab.ccmb.med.umich.edu/I-TASSER/).

### Recombineering experiments

Recombineering procedures were performed as previously described [18]. Briefly, *L. reuteri* LJO2 harboring pJP042 was induced to express RecT at OD_600_ between 0.55 and 0.65 for 20 min using 10 ng/ml of pSIP411 induction peptide. Cells were transformed by electroporation with 100 µg of recombineering oligo and 40 µg of oJP577, and recovered for 3 h in 1 ml of MRS. Cells were plated on MRS plates containing 25 µg/ml of rifampicin to select for cells that acquired the *rpo*B mutation targeted by the oJP577 oligomer. Recombineering oligonucleotides used for this work are listed in Additional file 5: Table S2, (IDT, USA) DNA was at a scale of 100 nm, desalted, without any modification. These oligonucleotides were designed as previously described, targeting the lagging strand of the gene to be mutated and introducing a stop codon as well as a restriction site [18]. Screening of the recombinant mutants was performed by PCR amplification of the targeted gene and digestion with the appropriate enzyme to confirm the mutation. The mutations were confirmed by Sanger sequencing.

### Prophage activity in LR 6475

Bacterial cultures were grown for 18 h, and subcultured to an OD_600_ of 0.01. At OD_600_ = 1, cultures were treated with Mitomycin C (Sigma Aldrich) (0.5 µg/ml) after which growth was monitored by measuring hourly the optical density. A reduction in OD_600_ in cultures subjected to Mitomycin C was indicative of prophage-mediated lysis.

### Statistical analysis

Student’s T-test and One way ANOVA followed by Fisher’s Least Significant Difference (LSD) post hoc test were used to determine whether differences among the groups were statistically significant (p < 0.05). Error bars indicate the standard deviation of the geometric mean.

## Additional files

**Additional file 1: Figure S1.** **a** *trg*A Basic Local Alignment Search Tool (BLAST) analysis ; **b** *trg*A and LreRMI locus structure.

**Additional file 2: Figure S2.** Improvements in transformation efficiency are linked to the type of plasmid transformed but not to the colony size. Transformation efficiency of small (S1 to S5) and big (B1 to B5) colonies, plasmid-cured from pNZ8048 (**a**) or pJP042 (**b**) and retransformed with pJP067 (red bars) or pNZ8048 (black bars) plasmids.   pNZ8048 was not evaluated in the strains plasmid-cured from pJP042. Data represent the averages of two independent experiments and error bars represent standard deviation.

**Additional file 3: Figure S3.** a. Growth curve; b. Phage induction with Mitomycin C.

**Additional file 4: Table S1.** Bacterial strains and plasmids used in this study.

**Additional file 5: Table S2.** Oligonucleotides used in this study.

## Abbreviations

LAB: lactic acid bacteria
LR 6475: *Lactobacillus reuteri* 6475
LreRMI: first type I restriction modification system in LR 6475
LreRMII: second type I restriction modification system in LR 6475
CR: conserved region
TRD: target recognition domain
*trg*A: transformation related gene A
